# A novel hybrid SEIQR model incorporating the effect of quarantine and lockdown regulations for COVID-19

**DOI:** 10.1038/s41598-021-03436-z

**Published:** 2021-12-15

**Authors:** R. Prabakaran, Sherlyn Jemimah, Puneet Rawat, Divya Sharma, M. Michael Gromiha

**Affiliations:** 1 Protein Bioinformatics Lab, Department of Biotechnology, Indian Institute of Technology Madras, Chennai, Tamil Nadu India; 2 Department of Computer Science, Tokyo Institute of Technology, Yokohama, Kanagawa Japan

**Keywords:** Computational biology and bioinformatics, Infectious diseases

## Abstract

Mitigating the devastating effect of COVID-19 is necessary to control the infectivity and mortality rates. Hence, several strategies such as quarantine of exposed and infected individuals and restricting movement through lockdown of geographical regions have been implemented in most countries. On the other hand, standard SEIR based mathematical models have been developed to understand the disease dynamics of COVID-19, and the proper inclusion of these restrictions is the rate-limiting step for the success of these models. In this work, we have developed a hybrid Susceptible-Exposed-Infected-Quarantined-Removed (SEIQR) model to explore the influence of quarantine and lockdown on disease propagation dynamics. The model is multi-compartmental, and it considers everyday variations in lockdown regulations, testing rate and quarantine individuals. Our model predicts a considerable difference in reported and actual recovered and deceased cases in qualitative agreement with recent reports.

## Introduction

The consequences of the COVID-19 pandemic on human life and the economy are disastrous, and the propagation of infection has not yet been controlled^1–4^. Governments have devised several strategies and imposed regulations and restrictions to decelerate the spread, control the cost of human lives and reduce the load on the health care industry^5–7^. While the development of several vaccines has been hopeful progress, evolving variants of SARS-COV-2 and its virulence is a threat^8–10^. Vaccination and acquired immunity have progressively led to the relaxation of lockdown restriction in a few geographical regions. However, considering the current vaccination rate and the bias in global distribution, most countries primarily rely on quarantine and lockdown procedures.

The quarantine and lockdown regulations and restrictions imposed by the governments target the reduction in disease transmission by confining the movement of the population and reducing the spread through human contacts^5,11,12^. Effective implementation of these procedures can slow down the spread and provide a window for the government to devise strategies to develop vaccines/drugs. Understanding the effect of lockdown on the dynamics of the pandemic is vital in planning and implementation^11^. SEIR model and its variants can be used in parameterizing and predicting the disease dynamics^13–16^. However, the typical formulations of the SEIR model do not take into account the complex effects of lockdown restrictions.

In the current study, we have adapted the standard SIER model to the current pandemic situation (COVID-19) by addressing specific essential observations such as the presence of asymptomatic carriers, reduction in transmission rate due to lockdown and its effect on the infection rate of the disease. By incorporating these parameters, we have developed a model to provide robust estimates of asymptomatic carriers in the population. Apart from providing an estimate of the infected and recovered population, these data would elucidate the role of these external factors on COVID-19 transmission. In addition, we have also incorporated the real-world, day-to-day mobility data, positive rate and number of tested samples into a Hybrid Susceptible-Exposed-Infected-Quarantined-Removed (HySEIQR) model. The model accounts for the effect of lockdown on disease transmission through inter-person contacts and the movement of people across geographic regions. Simulation of our detailed model showed a good correlation with the observed trend in the number of recovered cases.

## HySEIQR model, notations and assumptions

### Considerations in adapting SEIR model to COVID-19

Adapting standard SEIR model to the current scenario requires addressing the following key elements: (a) asymptomatic carriers, (b) effect of quarantine and lockdown, c) multi-compartmental approach, (d) testing rate and efficiency, (e) varying viral strains and their virulence, and (f) availability of medical resources and efficacy of treatment. These elements are known to strongly influence disease transmission rate (β), the spread of infected individuals to newer regions, and the recovery and mortality of patients.

COVID-19 infected individuals can be symptomatic or asymptomatic, and in most cases, can develop symptoms over time. However, statistical studies have shown varying proportions of symptomatic and asymptomatic cases in different populations^17,18^. In most countries, symptomatic and identified (tested) individuals are quarantined or advised to self-isolation. Isolation of infected cases removes them from the general population, thus reducing the spread of the disease. Further, identifying the infected cases depends directly on the testing rate in the region^19,20^.

### HySEIQR model

**Figure 1:**
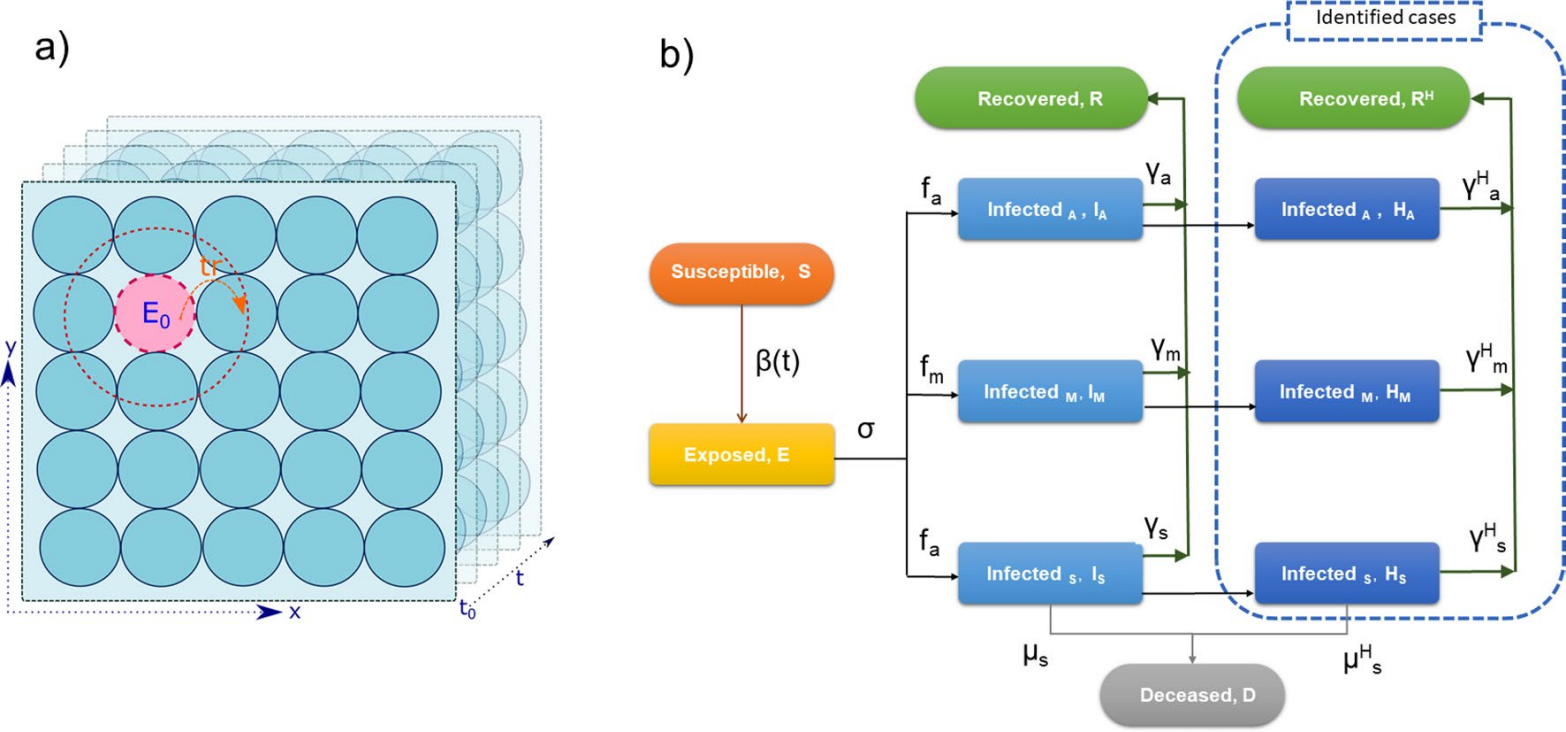
(**a**) Illustration of multi-compartmental approach in HySEIQR. Circles represent sub-regions or compartments. The movement of infected between the compartments/sub-regions is governed by the parameter Tr^Rate^. (**b**) the schematic representation of the Hybrid SEIRQ model (refer Eqs. 1–12).

The schematic representation of the SEIQR model is shown in Fig. 1, and the set of equations (Eqs. 1–10) listed below describes the model. Table 1 lists all the variables, parameters and constants along with the notations used in this study.

**Table 1:** List of variables, constants and parameters used in the model.

List of variables				
S. no	Variables	Notation		
1	Susceptible	S		
2	Exposed	E		
3	Infected	I		
4	Infected without symptoms	I_a_		
5	Infected with moderate symptoms	I_m_		
6	Infected with severe symptoms	I_s_		
7	Hospitalized/quarantined infected individuals without symptoms	H_a_		
8	Hospitalized/quarantined infected individuals with moderate symptoms	H_m_		
9	Hospitalized/quarantined infected individuals with severe symptoms	H_s_		
10	Recovered	R		
11	Recovered from disease without symptoms	R_a_		
12	Recovered from disease with moderate symptoms	R_m_		
13	Recovered from disease with severe symptoms	R_s_		
14	Recovered from disease without symptoms while hospitalized/quarantined	R_a_^H^		
15	Recovered from disease with moderate symptoms while hospitalized/quarantined	R_m_^H^		
16	Recovered from disease with severe symptoms while hospitalized/quarantined	R_s_^H^		
17	Deceased	D		
18	Deceased due to the disease with moderate symptoms	D_m_		
19	Deceased due to the disease with severe symptoms	Ds		
20	Susceptible and not infected individuals who were tested positive (false positives)	H_FP_		
21	Susceptible and not infected individuals who were positive after 14 days	R_FP_		

List of parameters				
S. no	Parameters	Notation	Value	Range
1	Disease transmission factor	β_0_	0.33	0.01–4
2	Initial number of exposed persons (t = 0)	E_0_	600	10–2000
3	Number of days between first exposed case and first identified case	lag	25	0–40
4	Lockdown coefficient for disease transmission (β)	W_β_	0.45	0.0–1.0
5	Number of sub-regions	N_e_	1000	1–5000
6	Lockdown coefficient for movement between sub-regions	W_T_	0.65	0.0–1.0
7	Transfer rate between sub-regions	Tr^Rate^_0_	0.25	0.0–1.0
8	Coefficient for disease transmission (β) due to asymptomatic cases	η	0.35	0.0–2.0

List of constants				
S. no	Constants	Notation	Value	
1	Number of repeats/runs of simulation	n	10	
2	Latent periods	1/σ	5	
3	Fraction of asymptomatic cases	f_A_	0.3	
4	Fraction of cases with moderate symptoms	f_M_	0.5	
5	Fraction of cases with severe symptoms	f_S_	0.2	
6	Recovery period for asymptomatic cases	1/γ_A_	7	
7	Recovery period for cases with moderate symptoms	1/γ_M_	10	
8	Recovery period for cases with moderate symptoms (hospitalized/quarantined)	1/γ_M_^H^	14	
9	Recovery period for cases with severe symptoms (hospitalized/quarantined)	1/γ_S_^H^	21	
10	Mortality rate for asymptomatic cases	μ_A_	0.0	
11	Mortality rate for cases with moderate symptoms	μ_M_	0.0001	
12	Mortality rate for severe symptomatic cases	μ_S_	0.0002	
13	Sensitivity of COVID-19 tests	Test TPR	0.90	
14	Specificity of COVID-19 tests	Test TNR	0.95	

1 $$\begin{array}{c}\dot{S}= -\beta \left(t\right).S*\left(\mathrm{Ia}.\upeta +\mathrm{Im}+\mathrm{Is}\right)\end{array}$$

2 $$\begin{array}{c}\dot{E}= \beta \left(t\right).S*\left(\mathrm{Ia}.\upeta +\mathrm{Im}+\mathrm{Is}\right)- \sigma E\end{array}$$

3 $$\begin{array}{c}\dot{{I}_{a}}= {f}_{a}.\sigma .E-\left({\mu }_{a}+{\gamma }_{a}\right).{I}_{a}-H\left({I}_{a}, t\right)\end{array}$$

4 $$\begin{array}{c}\dot{{I}_{m}}= {f}_{m}.\sigma .E-\left({\mu }_{m}+{\gamma }_{m}\right).{I}_{m}-H\left({I}_{m}, t\right)\end{array}$$

5 $$\begin{array}{c}\dot{{I}_{s}}= {f}_{s}.\sigma .E-\left({\mu }_{s}+{\gamma }_{s}\right).{I}_{s}-H\left({I}_{s}, t\right)\end{array}$$

6 $$\begin{array}{c}\dot{{H}_{a}}= H\left({I}_{a}, t\right)-\left({{\mu }_{a}}^{H}+{{\gamma }^{H}}_{a}\right).{I}_{a}\end{array}$$

7 $$\begin{array}{c}\dot{{H}_{m}}= H\left({I}_{m}, t\right)-\left({{\mu }_{m}}^{H}+{{\gamma }^{H}}_{m}\right).{I}_{m}\end{array}$$

8 $$\begin{array}{c}\dot{{H}_{s}}= H\left({I}_{s}, t\right)-\left({{\mu }_{s}}^{H}+{{\gamma }^{H}}_{s}\right).{I}_{s}\end{array}$$

9 $$\begin{array}{c}\dot{R}= {\gamma }_{a}.{I}_{a}+ {\gamma }_{m}.{I}_{m}+ {\gamma }_{s}.{I}_{s}+{{\gamma }_{a}}^{H}.{H}_{a}+ {{\gamma }_{m}}^{H}.{H}_{m}+ {{\gamma }_{s}}^{H}.{H}_{s}\end{array}$$

10 $$\begin{array}{c}\dot{D}= {\mu }_{a}.{I}_{a}+ {\mu }_{m}.{\mu }_{m}+ {\mu }_{s}.{I}_{s}+{{\mu }_{a}}^{H}.{H}_{a}+ {{\mu }_{m}}^{H}.{H}_{m}+ {{\mu }_{s}}^{H}.{H}_{s}\end{array}$$

where S, E, R and D denote the susceptible, exposed, recovered, and deceased population. $\sigma$, $\gamma$ and $\mu$ represent the incubation, recovery and mortality rate. Infected cases were grouped into three categories: asymptomatic (I_a_), moderately symptomatic (I_m_) and severely symptomatic (I_s_). Each category has a different recovery period (γ) and mortality rate (μ). And also, different contributions (η) to disease transmission (β). Similarly, H_a_, H_m_ and H_s_ represent the identified infected cases, including self-quarantined and hospitalized cases. The transmission of infection from Infected to Susceptible depends on the transmission factor, $\upbeta (\mathrm{t})$, a time-dependent variable. The number of hospitalized cases were obtained from real-world data (www.covid19India.org).

### Multi-compartment model and the stochastic nature

A typical epidemiological model assumes the region understudy to be a single compartmental with a homogenous density of exposed and infected cases across the region and throughout the time. One of the implications of lockdown restrictions is the localization by isolating sub-regions with a higher density of infected individuals. These restrictions create heterogeneity which requires a multi-compartmental approach. In the HySEIQR model, a country/state is uniformly divided into multiple sub-regions with boundaries. These regions are placed on a square map. The size and number of sub-regions depend on parameter N_e_ (number of compartments/sub-regions). The disease propagation dynamics is assumed to occur in each sub-region independently through the Eqs. (1–10). The movement of exposed and infected individuals between the neighbouring sub-regions is dependent on the transfer rate (Tr^Rate^_0_).

The inclusion of a multi-compartmental approach adds stochastic components to the model. The transfer of individuals from a sub-region to neighbouring sub-regions occurs through random selection of the neighbours. In addition, during the initialization of the simulation, the number of initially exposed individuals, E_0,_ is distributed among randomly selected sub-regions. These events vary with iterations due to their dependency on pseudo-random numbers.

### Incorporating real-world data into the model

The Hybrid SEIRQ model considers the day-to-day variations in government-imposed travel restrictions and lockdown conditions, the number of tested samples and positivity rate. The actual world data from various sources were collected and integrated into the model as functions λ(t), β(t) and H(t). We have collected the time-series data on the number of infections, deaths, recoveries, tested samples and positivity rate from COVID19India.org GitHub repository (https://api.covid19india.org) for the Indian population till May 2021. Further, the data on change in people movement was collected from Google mobility reports (https://github.com/GoogleCloudPlatform/covid-19-open-data) and Oxford stringency index^21^ (http://www.bsg.ox.ac.uk/covidtracker) as a measure of Quarantine and Lockdown stringency index (λ(t)). β(t) denotes the variation in transmission rate (Eq. 11). H(t) represents the actual number of positively tested cases in a day. The number of tested /identified cases predicted by the model on a day (t) depends directly on the number of infected cases (I_a_, I_m,_ and I_s_) on t but is limited by H(t). Seven-day window averages were used throughout our study to reduce non-specific day to day variations (Fig. S1).

11 $$\begin{array}{c}\beta \left(t\right)= {\beta }_{0}*\left({W}_{q}. (1-\lambda \left(t\right)) +\left(1-{W}_{q}\right) \right)\end{array}$$

12 $$\begin{array}{c}{\mathrm{Tr}}^{rate}\left(t\right)= {{Tr}^{rate}}_{0}*\left({W}_{T}. (1-\lambda \left(t\right)) +\left(1-{W}_{T}\right) \right)\end{array}$$

where W_q_ and W_T_ denote the weight associated with lockdown regulations, λ(t).

## Interpreting the HySEIQR model

### Parameter estimation and fit

The parameters for the model were either defined as parameters (Table 1) and estimated through non-linear data fitting or as constants and derived from literature and published reports (Table 1). The parameters associated with disease dynamics, such as σ, β and γ, have been well studied and declared constants in this work^22–24^.

The rest of the parameters were estimated through a two-step approach. A systematic grid search is performed in the parameter space (Table 1). For each point in the grid, the least-square optimization algorithm (least_squares) was applied to minimize the least-squares error between the predicted and actual number of identified recovered and infected cases^25,26^. The model with the lowest error was selected and further optimized.

### Comparison between predicted and actual data

**Figure 2:**
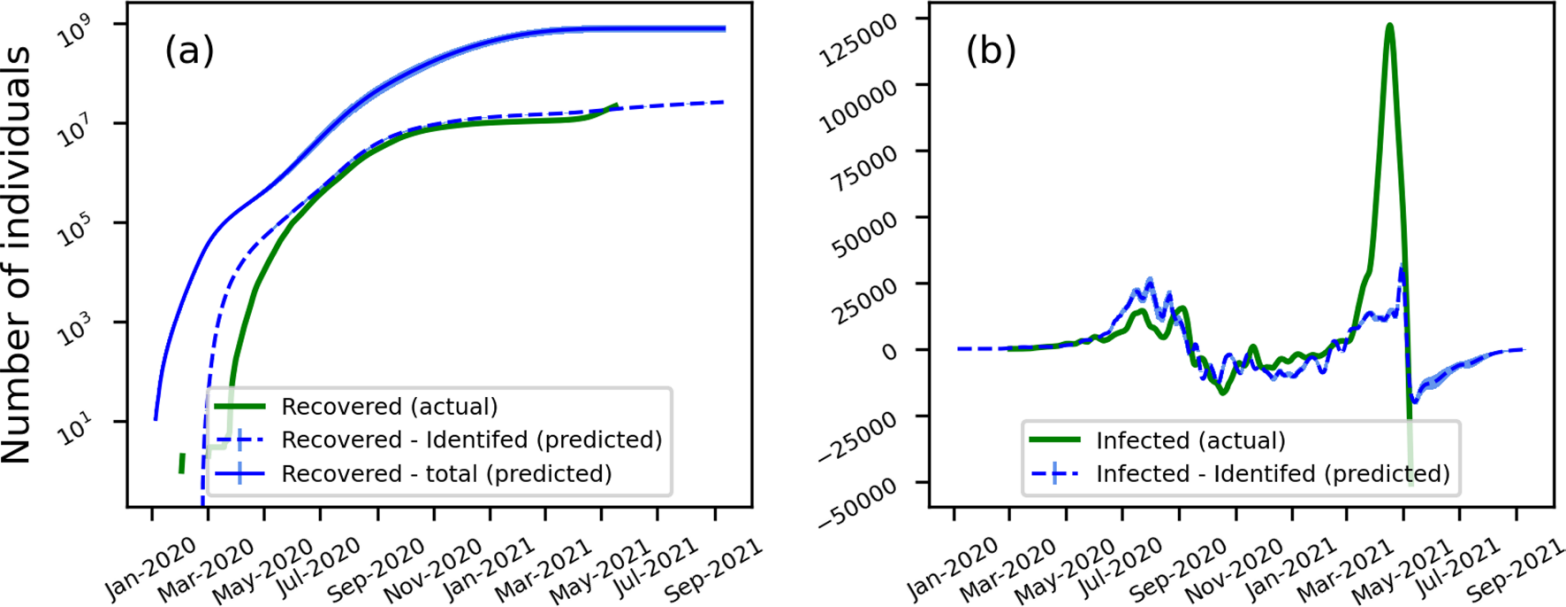
Actual and predicted number of (**a**) recovered and (**b**) change in infected COVID-19 cases in India. The shaded regions represent the standard error from 10 replicates.

Figure 2 shows the predicted number of recovered cases and change in infected cases over time, along with the actual data for the Indian population. As of 15th May 2021, approximately 20.82 million and 270,000 recovered and deceased as per the available data with 3.54 million existing infected cases. Our model predicted 18.56 million identified and 772.28 million unidentified recovered cases. The number of unidentified recovered cases is nearly 37 [25, 49] times higher than the reported number of recovered cases. Similarly, our model predicted 2.6 [1.7–3.5] times higher deceased cases than reported deaths due to COVID-19. In other words, on average only ~ 3 in 100 recovered cases and ~ 1 in 3 deaths is reported. Despite the variations in the prediction results over iterations and its high sensitivity to parameters, the model consistently indicates several times higher recovered cases than reported. These results agree with earlier reports of undetected COVID-19 cases^27–29^. Especially considering the asymptomatic cases and the low testing rate, the actual number of recovered cases can be several times higher than the reported numbers.

**Figure 3:**
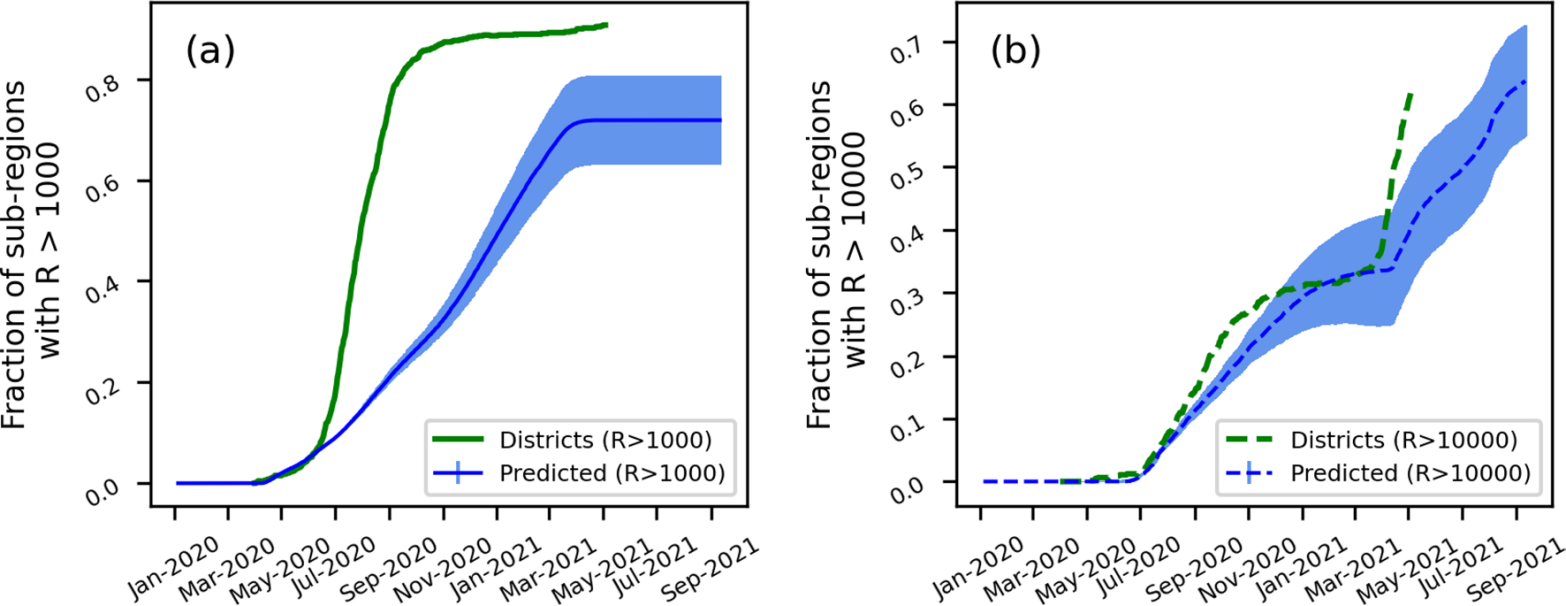
Comparing the predictions for the sub-regions in our model with the actual data from Indian districts. The y-axis represents the fraction of sub-regions (blue line)/districts (green line) with (**a**) more than 1000 recovered cases and (**b**) more than 10,000 recovered cases. The shaded regions represent the standard error from 10 replicates.

To validate our simplified multi-compartmental design, we compared the distribution of recovered cases across the compartment/sub-regions with relevant administrative sub-division in India. We chose districts for comparison since our model consists of 1000 sub-regions in the similar range as the number of districts in India. Figure 3 shows the fraction of sub-regions and districts with the number of recovered cases greater than 1000 and 10,000. A threshold of 1000 was used to check the presence of COVID-19 infection in a sub-region. In addition, a threshold of 10,000 was chosen to test the widespread of the disease. The results indicate that our model underestimates the presence of COVID-19 infection at a threshold of 1000). However, the model showed a better correlation with the spread of the disease in sub-regions at a threshold of 10,000. The difference between the predicted (sub-regions) and actual (districts) is expected since the size and distribution of sub-regions are uniformly modelled, whereas districts vary significantly in size and geographical locations. The gaps can be overcome by using a network topology based on the distribution of districts sizes and their connectivity^30,31^.

### Influence of lockdown regulations on disease-control

**Figure 4:**
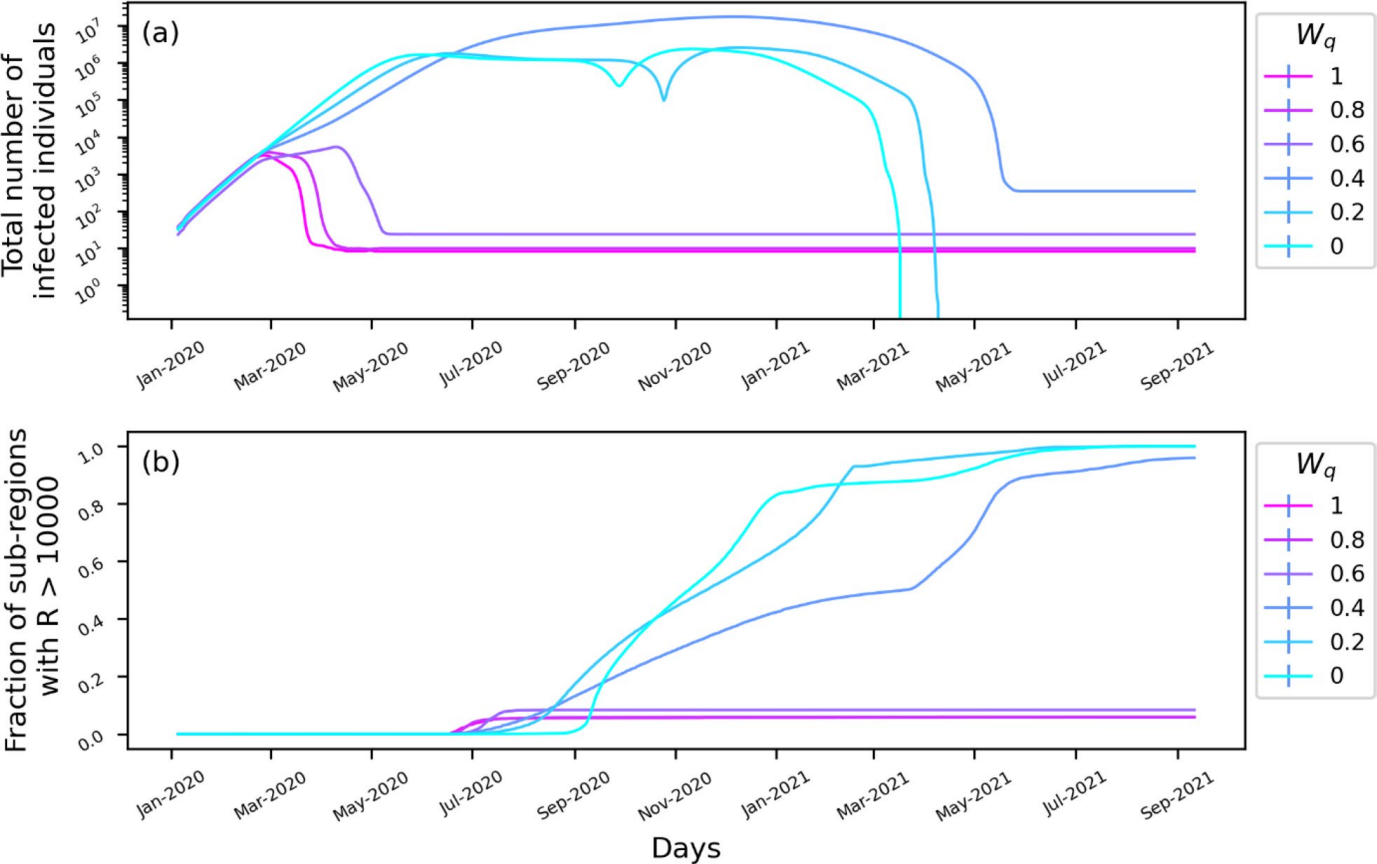
Effect of lockdown and quarantine on the spread of COVID-19 infection. The simulated change in the (**a**) total number of infected individuals (identified and unidentified) and (**b**) the number of sub-regions/compartments with more than 1000 recovered cases as a function of the parameter, Wq.

**Figure 5:**
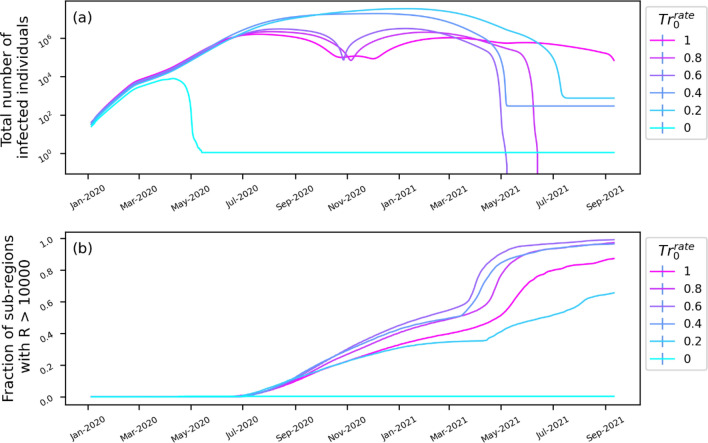
Role of inter-compartment movement on the spread of COVID-19 infection. The simulated change in the (**a**) total number of infected individuals (identified and unidentified) and (**b**) the number of sub-regions/compartments with more than 1000 recovered cases as a function of the parameter, Tr^Rate^_0_, transfer rate between sub-regions.

The lockdown-imposed restrictions (λ(t)) affects the disease transmission factor (β_0_) as described in Eq. 11. W_q_ is the weight that determines the influence of the λ(t) on β. Higher W_q_ indicates a more substantial influence of lockdown rules in reducing disease transmission. To study the influence of the parameter, we run simulations by varying W_q_ from 0 to 1 at intervals of 0.2 (Fig. 4). Figure 4a shows the change in the total number of infected cases (identified and unidentified) over time. The results show that with strong adherence to government-imposed lockdown regulations (W_q_ ≥ 0.6), the spread of COVID-19 could have been controlled within six months. However, non-adherence (W_q_ ≤ 0.4) to the restriction could lead to the rapid spread of disease with an average of million cases per day and spreading to almost every sub-region (Fig. 4b). We repeated the analysis by varying Tr_0_^rate^, the transfer rate of infected between sub-regions (Fig. 5). With zero movements between the sub-regions (Tr_0_^rate^ = 0), the spread of disease is restricted to only the initial compartments and spread to compartments is stopped. Increasing Tr_0_^rate^ increases the rate of spread to other sub-regions, leading to a rapid increase in infection rate.

## Discussion

Several methods have been developed using modified SEIR models to understand the spread of COVID-19. The most necessary adaptions are (i) identification of infected cases and quarantine of suspected cases, (ii) role of lockdown on social interactions and movement of the population and (iii) inclusion of asymptomatic cases^32–34^. Quarantine is one of the important strategies in controlling any contagious disease^32^. ﻿Senapati et al.^35^ developed a deterministic compartmental model incorporating quarantined and hospitalized for mild and severe symptomatic cases, respectively. The rate of quarantine and hospitalization are usually determined as part of the model parameters. In our model, the quarantine/hospitalization is determined by the number of actual tested cases in the region obtained from real-world data and fed to the model. These tested cases are further distributed among symptomatic and asymptomatic cases based on testing sensitivity and specificity. These suspected cases, which include true and false positives, are quarantined during the expected recovery period (determined by γ). These assumptions are close to a real-world situation and also easily adaptable to other geographical regions.

The effect of lockdown on disease transmission is time-dependent and complex with various direct and indirect influences. For example, the government imposed regulations directly create barriers for the movement of people and indirectly generate awareness among the population to follow hygienic practices. Although these actions reduce the disease transmission rate (β), the influence of these measures changes over time. To accommodate this effect, studies have modelled β as a function of time or lockdown^36–38^. Ianni and Rossi^36^ represented β as a decreasing exponential function to accommodate the increasing awareness of the population and the reducing disease transmission rate over 120 days since disease outbreak.

On the other hand, the awareness could gradually reduce over an extended period and governments can impose lockdown in phases, which create waves of awareness. While such approaches are convenient and easily adapted to standard epidemiological models/equations, disease transmission rate depends on complex social interactions among the population/community. Networks/graphs representing the interaction pattern among communities are often used to overcome these shortcomings^30,31,39,40^. In our model, we have incorporated two measures, namely Google mobility reports (https://github.com/GoogleCloudPlatform/covid-19-open-data) and Oxford stringency index (Hale et al., 2021) (http://www.bsg.ox.ac.uk/covidtracker) as a measure of Quarantine and Lockdown stringency index (λ(t)). These measures represent the dynamic changes in government norms and associated population behaviour towards COVID-19. Thus, it provides a better reflection of the real-world situation for the model. The λ(t) influences the disease transmission rate, β and also affects the movement of people from one region to another, Tr^rate^ in the model (Eqs. 11 and 12). In addition, the multi-compartmental design model raises a barrier to people moving from one sub-region to another. Thus, the model mimics the effect of lockdown in a large geographical region like India.

Consideration of asymptomatic cases is another crucial and essential criterion for a COVID-19 epidemiological model. Models, which incorporate asymptomatic cases consider a part of the infected cases to be asymptomatic with no identifiable symptoms and are probably undetected. This fraction of the infected patients undergoes natural recovery over a period of time^27,41,42^. A similar approach is employed in our model. Infected cases are considered to be part of one of the three classes: (i) asymptomatic, (ii) symptomatic with moderate and (ii) symptomatic with severe symptoms. Few models treated a constant fraction of infected cases as asymptomatic, which is determined by model optimization. These asymptomatic cases can remain asymptomatic until recovery or may show symptoms over time^42^.

## Conclusions

We have developed a hybrid SEIQR model by incorporating several adaptations for COVID-19 disease, testing protocols, current quarantine, and lockdown regulations. In our approach to the model, several assumptions and simplifications were imposed to account for the following: (i) The government imposed lockdown regulations were represented through over-simplified metrics from openly available reports, (ii) The role of hospitals in controlling mortality rate, allocation and availability of equipment in hospitals, the effect of viral strains in disease transmission and mortality rate were not factorized into the model and (iii) only part of the parameters was optimized, and the rest were considered constants based on the literature to ease parameter optimization.

Despite the limitations, our model captured the essence of the quarantine, lockdown and movement of infected between the regions. The model was developed with minimal dependency using core python libraries and is available as a webserver at https://web.iitm.ac.in/bioinfo2/covid19hyseiqr/home. The model is highly customizable and can be adapted to further modifications. The inclusion of network topology of administrative divisions in India and the effects of viral strains would benefit the community to a greater extent.

### Ethics declarations

No experiments on Human or Animals were conducted as part of the study. All data used in the study were collected from openly available repositories.

## Supplementary Information

Supplementary Information.

## Data Availability

All data used in the study were collected from openly available repositories. Sources are listed in the manuscript. In addition, the model is available as a webserver at https://web.iitm.ac.in/bioinfo2/covid19hyseiqr/home.
