# Supplementary material for: A novel hybrid SEIQR model incorporating the effect of quarantine and lockdown regulations for COVID-19
Source: Sci Rep. 2021 Dec 15;11:24073. doi: 10.1038/s41598-021-03436-z (PMC8674241; doi:10.1038/s41598-021-03436-z)

**A novel hybrid SEIQR model incorporating the effect of quarantine and lockdown regulations for COVID-19**

R. Prabakaran^1^, Sherlyn Jemimah^1^, Puneet Rawat^1^, Divya Sharma^1^ and M. Michael Gromiha^1,2,*^

^1^Protein Bioinformatics Lab, Department of Biotechnology, Indian Institute of Technology Madras, Chennai, Tamil Nadu, India

^2^Department of Computer Science, Tokyo Institute of Technology, Yokohama, Kanagawa, Japan

**Supplementary Information**

------------------------------------

Corresponding author:

MMG: [gromiha@iitm.ac.in](mailto:gromiha@iitm.ac.in)

**Supplementary Information**


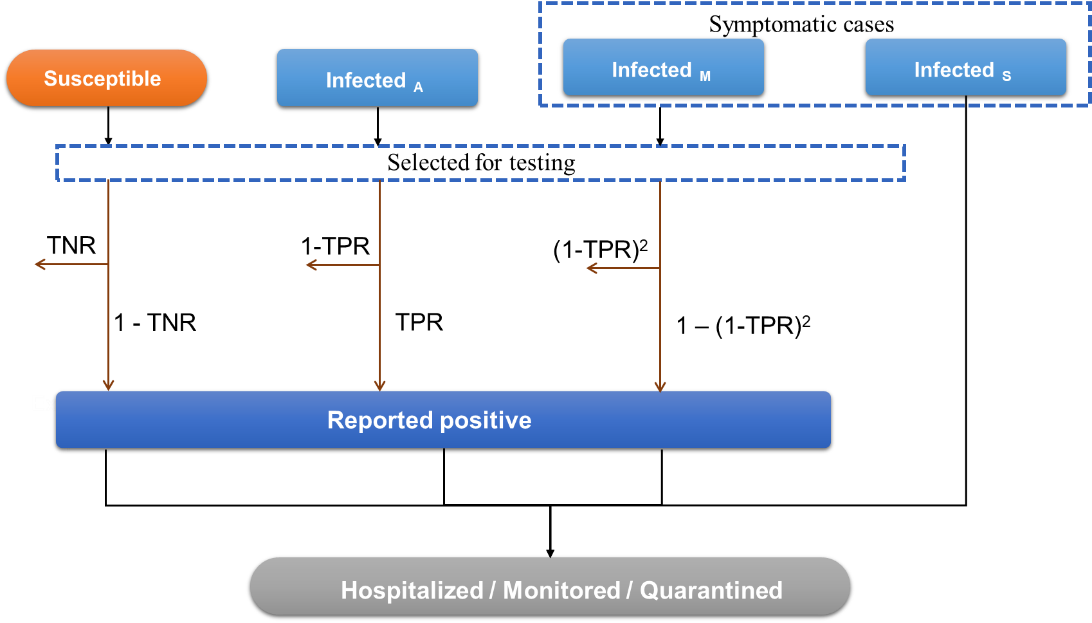


**Figure S1**: Identification of positive individuals through COVID-19 tests. The sensitivity and specificity of the test were assumed to be constants at 0.90 and 0.95, respectively. However, the values can change depending on various parameters (Online references: <https://www.finddx.org/covid-19/sarscov2-eval-molecular/molecular-eval-results/>, https://www.icmr.gov.in/cteststrat.html )

**
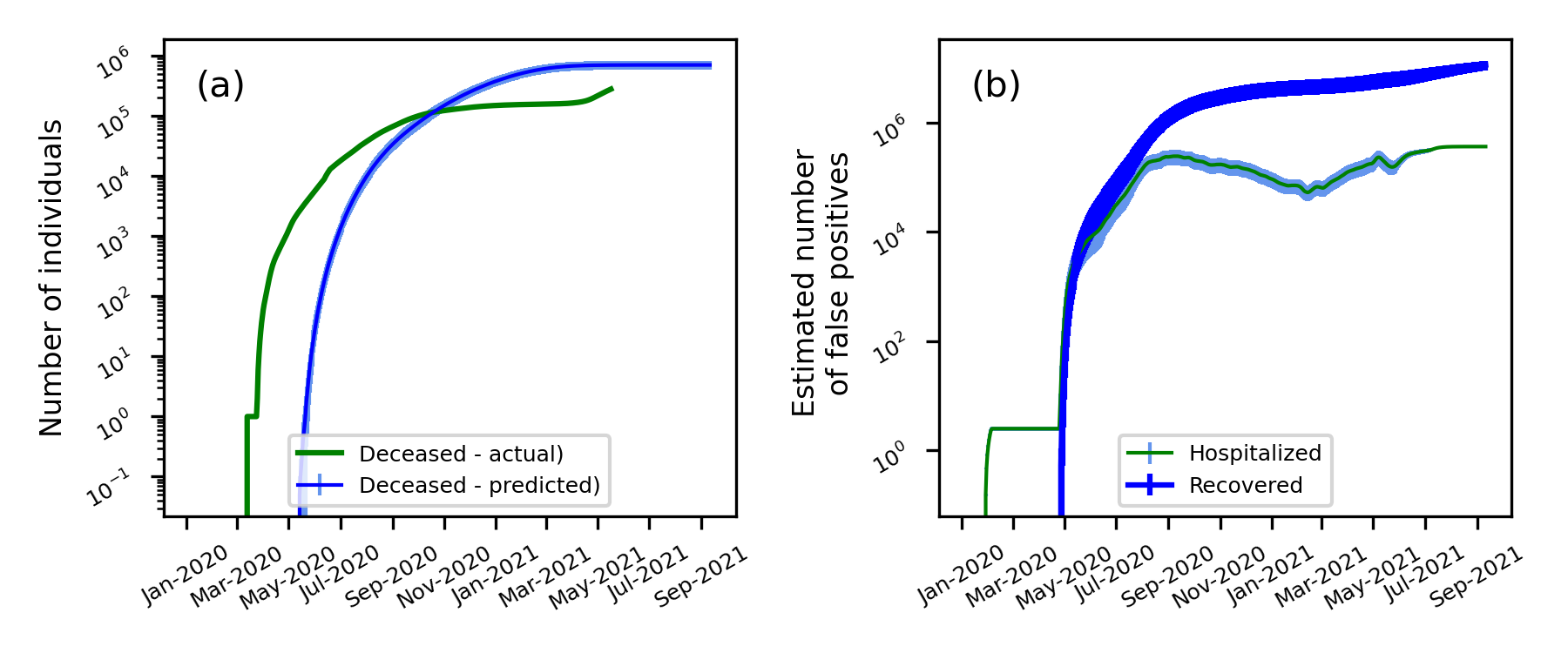
**

**Figure S2:** Predictions from HySEIQR: (a) the actual and predicted number of deceased individuals (b) number of healthy individuals predicted to be tested positive and recovered

**Figure S3:** Screenshots of the webserver. (<https://web.iitm.ac.in/bioinfo2/covid19hyseiqr/home>). Home page showing prediction output from HySEIQR for India through an interactive graphical interface and (b) panel to reconfigure the model by changing the parameters and constants used in the model


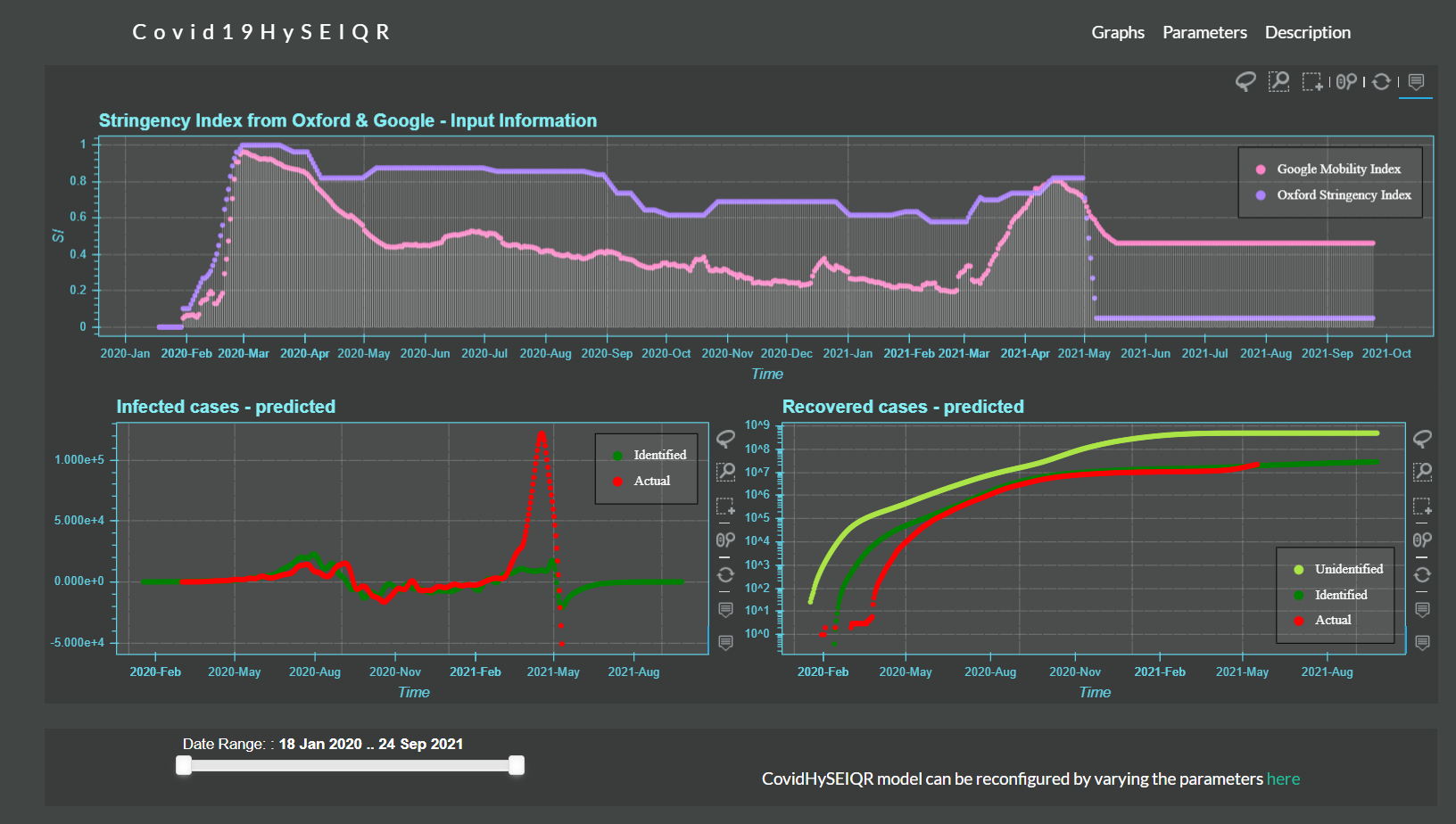


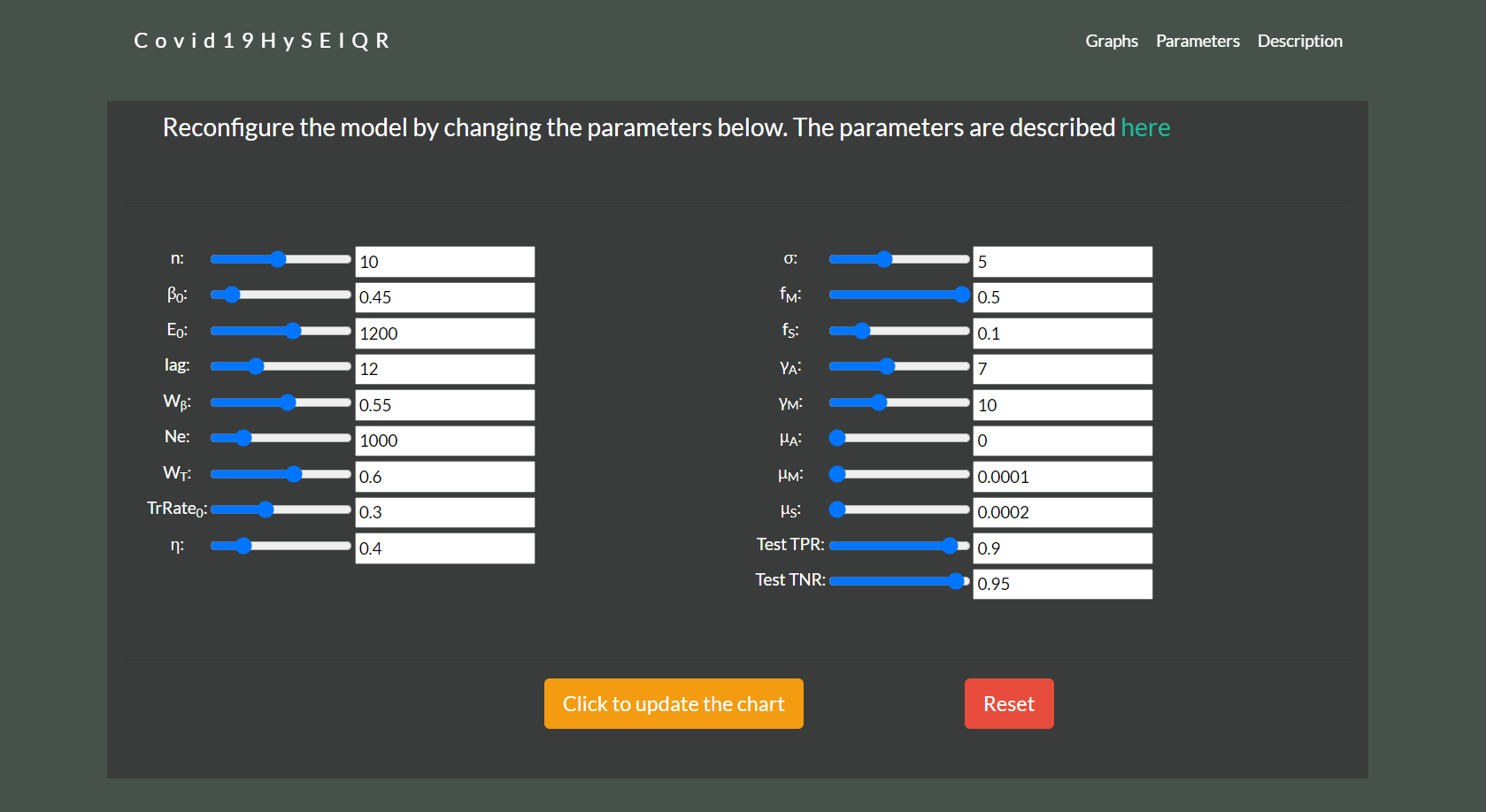

Supplement: Supplementary file 1 — Supplementary Information. [file 41598_2021_3436_MOESM1_ESM.docx]
